# Using normalisation process theory for intervention development, implementation and refinement in musculoskeletal and orthopaedic interventions: a qualitative systematic review

**DOI:** 10.1186/s43058-023-00499-z

**Published:** 2023-09-18

**Authors:** Hayley Carter, David Beard, Alison Harvey, Paul Leighton, Fiona Moffatt, Benjamin Smith, Kate Webster, Pip Logan

**Affiliations:** 1 Physiotherapy Outpatients, Level 3, Florence Nightingale Community Hospital, University Hospitals of Derby and Burton NHS Foundation Trust, Derby, DE1 2QY UK; 2 School of Medicine, University of Nottingham, Queens Medical Centre, Nottingham, NG7 2UH UK; 3 Surgical Intervention Trials Unit, Botnar Research Centre, NDORMS, University of Oxford, Windmill Road, Oxford, OX3 7LD UK; 4 School of Health Sciences, University of Nottingham, Queen’s Medical Centre, Nottingham, NG7 2HA UK; 5 Health Sciences 3 Building, La Trobe University, Kingsbury Drive, Bundoora, VIC 3086 Australia

**Keywords:** Normalisation process theory, Extended normalisation process theory, NoMAD, Orthopaedic conditions, Musculoskeletal conditions, Complex interventions

## Abstract

**Background:**

Normalisation process theory (NPT) provides researchers with a set of tools to support the understanding of the implementation, normalisation and sustainment of an intervention in practice. Previous reviews of published research have explored NPT’s use in the implementation processes of healthcare interventions. However, its utility in intervention research, specifically in orthopaedic and musculoskeletal interventions, remains unclear. The aim of this review is to explore how NPT (including extended NPT, ENPT) has been used in orthopaedic/musculoskeletal intervention research.

**Methods:**

A qualitative systematic review was conducted. Two bibliographic databases (Scopus and Web of Science) and a search engine (Google Scholar) were searched for peer-reviewed journal articles citing key papers outlining the development of NPT, related methods, tools or the web-based toolkit. We included studies of any method, including protocols, and did not exclude based on published language. A data extraction tool was developed, and data were analysed using a framework approach.

**Results:**

Citation searches, of the 12 key studies, revealed 10,420 citations. Following duplicate removal, title, abstract and full-text screening, 14 papers from 12 studies were included. There were 8 key findings assessed against GRADE-CERQual (Confidence in Evidence from Reviews of Qualitative research). Five were of high confidence supporting NPT/ENPT’s use in the implementation process for interventions targeting a range of MSK/orthopaedic conditions. NPT/ENPT offers a useful analytical lens to focus attention and consider implementation factors robustly. There is limited evidence for the selection of NPT/ENPT and for the use of the Normalisation Measure Development instrument. Three findings of moderate confidence suggest that coherence is seen as a fundamental initial step in implementation, there is limited evidence that study population limits NPT’s utility and the application of ENPT may pose a challenge to researchers.

**Conclusion:**

This review demonstrates NPT’s utility in supporting intervention implementation for orthopaedic and musculoskeletal conditions. We have theorised the benefits ENPT offers to intervention development and refinement and recommend future researchers consider its use. We also encourage future researchers to offer clear justification for NPT’s use in their methodology.

**Trial registration:**

The review protocol is registered with PROSPERO (CRD42022358558).

**Supplementary Information:**

The online version contains supplementary material available at 10.1186/s43058-023-00499-z.

Contributions to the literature

- To date, research has demonstrated the use of the normalisation process theory in helping to understand how healthcare interventions work in practice.
- This review adds to the body of knowledge, supporting normalisation process theory’s use to understand how interventions work in research specific to orthopaedic and/or musculoskeletal conditions, such as knee replacements and lower back pain.
- Future researchers are encouraged to offer further description as to why specific parts, or versions of the normalisation process theory are chosen and consider offering feedback on their experience of using the theory in their research project.

## Introduction

The development and evaluation of complex interventions in healthcare has been supported by the Medical Research Council (MRC) guidance since the first iteration in 2000 [1], subsequently revised in 2006 [2] and 2021 [3]. The 2021 framework provides further consideration as to how interventions interact within the context they are implemented to bring about change. It divides complex intervention research into four phases, underpinned by six core elements, and promotes intervention research as an iterative cycle that may begin at any phase [3].

To compliment the MRC framework, O’Cathain and colleagues offer additional guidance [4] for consideration during intervention development. Two key actions highlighted in this guidance which support a recognised approach to intervention development (implementation focused) [5] are to ‘draw on existing theory’ and ‘pay attention to future implementation of the intervention in the real world’; it is therefore of no surprise that implementation science theories have been an area of interest to developers. There are a number of theories, outlined in the narrative review by Nilsen in 2015 [6], that function to prioritise aspects related to ‘the how and why’ of implementation. The normalisation process theory (NPT) is a popular choice among healthcare researchers with one of the key papers outlining the theory being cited over 500 times in Scopus [7]. There is no one theory identified to be superior to another and researchers have previously commented on the difficulties with choosing an appropriate approach [6, 8, 9]. NPT is perhaps popular in the field of health science, because of its developer’s encouragement that there is ‘no right way to employ NPT’ [10].

### Normalisation process theory

In its first iteration, NPT was an applied theoretical model, known as the normalisation process model (NPM), developed by Carl May and colleagues in 2006 [11, 12]. NPM aimed to facilitate the understanding and evaluation of factors that facilitate or inhibit the routine integration of complex healthcare interventions in practice [13]. Empirical application identified NPM’s utility in explaining factors related to ‘collective action’ (the work participants do to make the intervention work).

NPT was later developed in 2009 [14], extending the model to a middle-range theory as it was acknowledged that NPM had limited scope to explain factors beyond collective action. This development led to three further constructs of coherence (meaning and sense-making), cognitive participation (commitment and engagement) and reflexive monitoring (reflection or appraisal) [14]. NPT therefore provides researchers with a set of tools to support the understanding of the implementation, normalisation and sustainment of an intervention in practice [15].

**Fig. 1:**
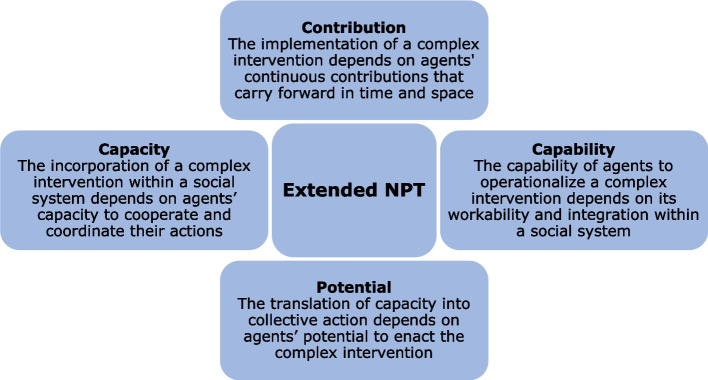
Extended normalisation process theory

In more recent years (2013 onwards), the theory developed further to pay attention to dynamic implementation contexts [16, 17]. Four further constructs were defined by May in 2013, described as the ‘extended normalisation process theory’ (ENPT) [16], shown in Fig. 1.

ENPT aims to provide a more detailed explanation of the implementation process by describing interactions between (1) agency (i.e., the work people do and the ways they work with components of the complex intervention) and (2) context (the resources people draw on to realise agency) [16].

**Fig. 2:**
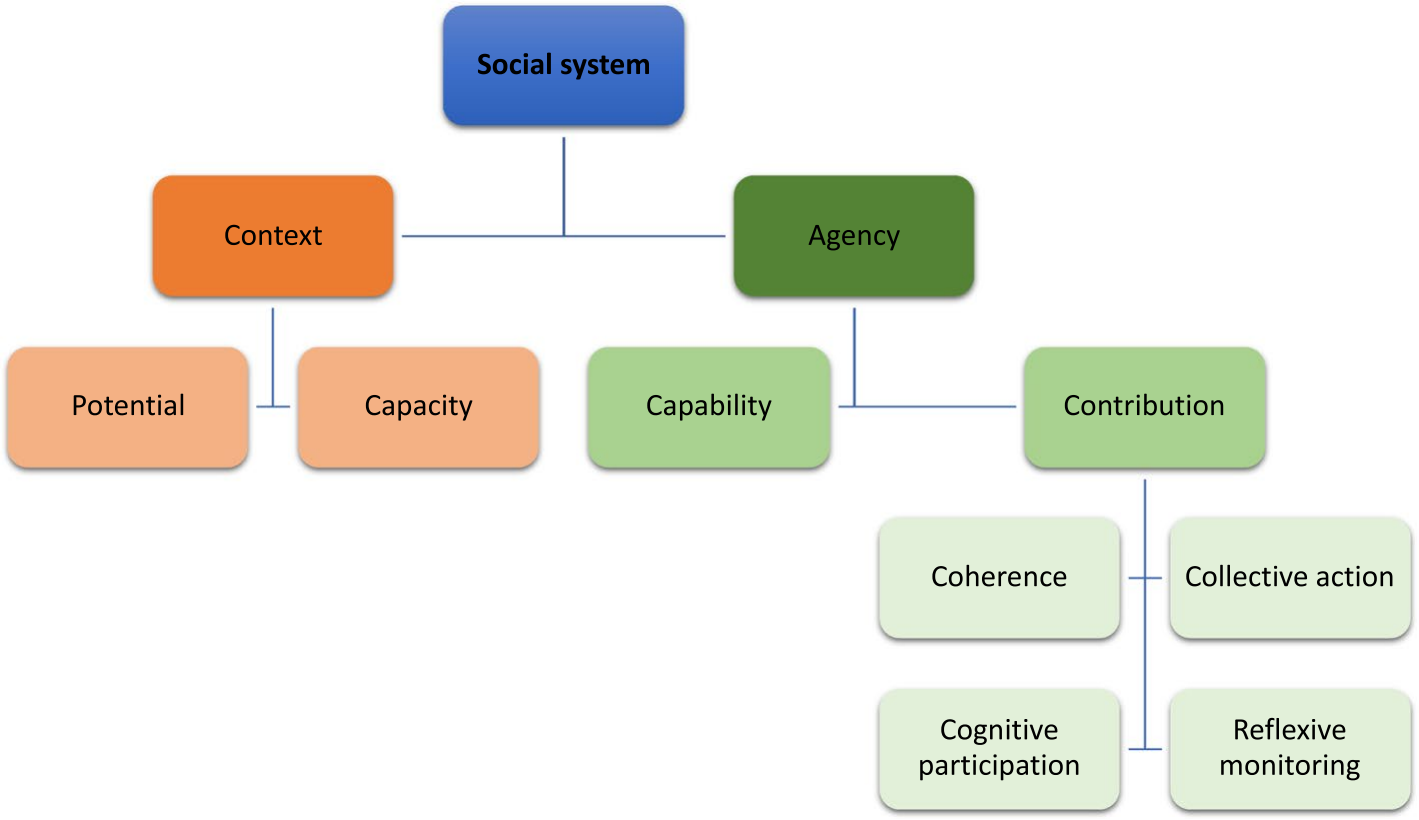
Organisation of ENPT and NPT. Adapted from Fig. 2 ‘Towards a general theory of implementation’ [16]

The constructs of capability and contribution sit within the bracket of agency and the constructs of potential and capacity sit within context. The constructs of the 2009 version of NPT subsequently sit within ‘contribution’ and thus focus purely on expressions of agency independent of the context (shown in Fig. 2).

Another final tool available for use is the Normalisation Measure Development (NoMAD), a 23-item instrument developed in 2018, based on NPT, to support the measurement of implementation factors affecting normalisation [18]. The NoMAD’s specific focus is on the participants’ (those delivering or receiving it) experiences of implementation. It was suggested by the developers to be viewed as a pragmatic measure that could be applied flexibly to meet research and practice needs [19].

### Purpose of this review

A review by May et al. [10] outlined the uses and limits of NPT in the implementation of healthcare interventions in addition to exploring its contribution to the dynamics of these processes. In this review, one study (*n* = 1/130) was categorised to use NPT in ‘intervention design’. A recent review by Huddlestone and colleagues [20], exploring the application of NPT in implementation processes in a UK primary care setting, found only five studies (*n* = 5/35) using NPT as a framework for ‘intervention development’. However, two of the five studies included in Huddlestone et al.’s review were categorised differently by May et al., identified to use NPT as a tool for ‘organisation and delivery’ rather than ‘intervention design’. The terminology used also differed between the two reviews, with May et al. referring to ‘intervention design’ and Huddlestone et al. to ‘intervention development’. It is therefore clear that the language used to describe intervention development and implementation procedures amongst healthcare literature is open to interpretation.

What remains unclear, is how NPT has been used in intervention research specific to the context of orthopaedic and musculoskeletal (MSK) conditions. Over 20 million people in the UK live with an MSK condition [21] and over 750,000 patients currently sit on an NHS orthopaedic waiting list in England [22, 23]. The development of complex interventions targeted at improving the care and outcomes of this patient group is therefore an important area of healthcare research and aligns with several research priorities [24–27].

Complex intervention development is resource-intensive. Careful consideration of the approach is vital to ensure rigour and, therefore, cost-effectiveness. Understanding the utility of NPT in intervention research specific to orthopaedic and MSK conditions may inform its future use in similar research, in particular intervention development. Knowledge of NPT’s usability with an understanding of barriers and facilitators to its use will also support future researchers by offering clarity to NPT’s use whilst ensuring previous pitfalls are not repeated.

The purpose of this review is to explore how NPT has been used in intervention research targeting adults (≥ 18 years old) with an orthopaedic and/or MSK condition in a healthcare setting. The objectives are to (1) identify how NPT has been used (e.g. in the process of intervention development, implementation and/or refinement), (2) explore insight generated in the use of NPT and (3) understand the benefits and disadvantages of using NPT as critiqued by researchers.

### Definitions used in this review

Some definitions are used interchangeably among the literature to define intervention development processes. For the purpose of this paper, we have defined our interpretation of the following terms in Table 1.

**Table 1:** Definitions used in this paper

Term	Definition
Development	Any process related to the inception and design of a novel intervention. This can be anywhere on the continuum from initial scoping work, to determine the need for the intervention, through to the developed prototype
Implementation	The process of embedding the intervention within the setting/context of its intended use
Refinement	The process of refining or adapting an already existing intervention

## Methods

### Reporting and registration

This review is reported according to the ENTREQ (Enhancing transparency in reporting the synthesis of qualitative research) statement (Additional file 1) [28] and registered with PROSPERO (CRD42022358558, https://www.crd.york.ac.uk/prospero/display_record.php?ID=CRD42022358558).

### Search strategy

The search strategy outlined in the protocol was followed. Similar to the strategy outlined by May et al. [10] and Kirk et al. [29], the main method of searching was focused on citations. Citations of key papers outlining the development of NPT, related methods or tools in addition to the web-based toolkit (Additional file 2) were searched in two bibliographic databases (Scopus and Web of Science) and a search engine (Google Scholar) from inception to November 2022. The keywords ‘orthopaedic’ and ‘musculoskeletal’ were applied to narrow the results.

### Eligibility criteria

An overview of the inclusion and exclusion criteria is shown in Table 2.

**Table 2:** Eligibility criteria

Inclusion	Exclusion
• Peer-reviewed journal articles where NPT was used in the intervention development, implementation or refinement process • Any healthcare setting • Any intervention targeted at an orthopaedic or musculoskeletal condition, e.g. exercise, leaflet, website and screening tool • Any method, including protocols, provided there was a clear explanation of how NPT will be or has been used • Any study that used NPT as a stand-alone theory or in combination with other theories (a clear description must be provided for NPT’s specific contribution to the process where it is used alongside other theories) • Any language where it is feasible and pragmatic to translate the article into English. (In the first instance, Google Translate will be used for title and abstract screening. Where a study is included for full-text screening, the translation will be checked, where possible, with a native speaker of the language via Cochrane Task Exchange or colleagues within the University of Nottingham)	• NPT influence not clearly explained • Intervention implemented outside of a healthcare setting • Intervention not targeted at an orthopaedic or musculoskeletal condition

### Data extraction

Data were extracted by two reviewers (HC and AH) using an instrument developed by the review team. Data were extracted on: authors, year of study, study type, population/study setting/condition, intervention, use of NPT (intervention development, implementation or refinement), insight generated in the use of NPT and author critique of NPT.

### Quality appraisal

Quality appraisal of included articles was carried out using the CASP checklist [30] with guidance from the Cochrane Handbook (chapter 21) [31] and Long et al. [32].

### Data analysis

**Fig. 3:**
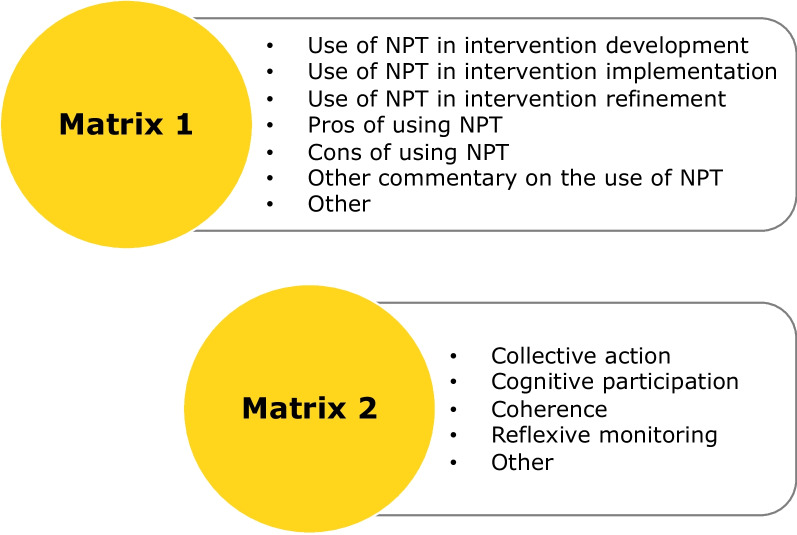
Provisional matrices

Framework analysis was used to identify the use and commentary of NPT in orthopaedic/MSK intervention research in addition to exploring insight generated in the use of NPT. Two matrices were developed a priori, as described in the protocol, with matrix one mapping to objectives (1) and (3) and matrix two mapping to objective (2) (Fig. 3).

**Fig. 4:**
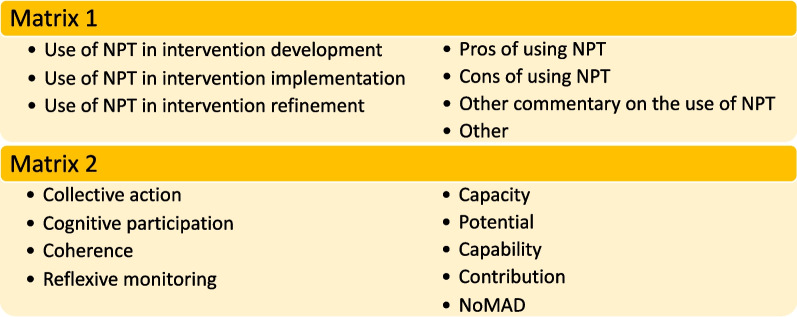
Final matrices

Relevant data were imported, from all sections of the paper, into Microsoft Excel (Microsoft Cop., Redmon, WA, USA) for analysis. Initial analysis focused on matrix one, aiming to understand and identify how NPT had been used in each study. The a priori headings aligned with the review objectives and no refinement was required. In matrix two, the pre-defined headings included the constructs of collective action, cognitive participation, coherence and reflexive monitoring. However, four of the 14 papers identified the constructs of ENPT and so the matrix was expanded to include capacity, potential, capability and contribution. One study also utilised the NoMAD questionnaire and so this was also included as an item in the matrix. After this initial mapping to matrix two, charting was completed for 25% of the dataset. The items were reviewed and felt to cover all aspects of NPT’s use and the remainder of the dataset was mapped to the refined matrices (Fig. 4).

Following data mapping onto the two matrices, data were organised into broad themes with the aim to summarise the dataset: (1) What was NPT used to support?; (2) NPT use, justification and insight generated; and (3) critique and commentary of NPT use.

### Assessment of confidence

GRADE-CERQual (Confidence in the Evidence from Reviews of Qualitative research) was used to assess the confidence of the review findings [33]. The interactive Summary of Qualitative Findings (iSoQ) tool was used to facilitate its application [34] in addition to guidance from the GRADE-CERQual paper series [35–39].

## Results

### Search results

**Fig. 5:**
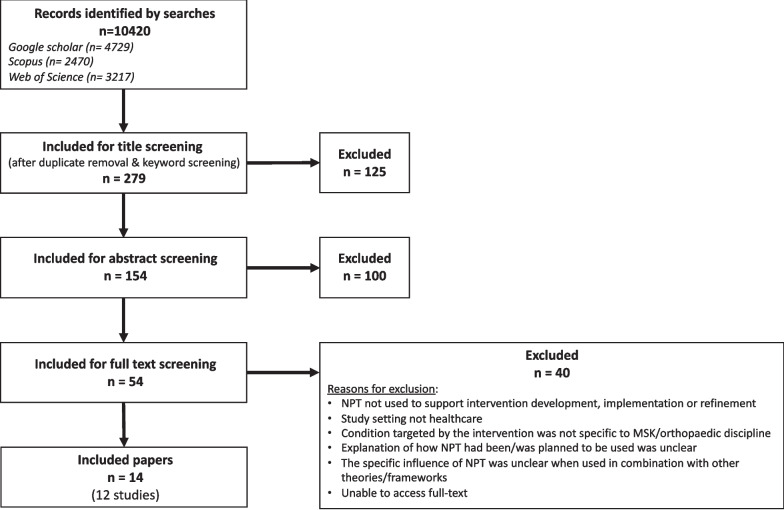
PRISMA flow diagram

Citation searches, of the 12 key studies, in the two databases and search engine revealed 10,420 citations. A PRISMA flowchart is shown in Fig. 5. Keyword searching of ‘musculoskeletal’ and ‘orthopaedic’ narrowed the results to 755 citations. Following duplicate removal, 279 titles were screened for inclusion. Of these, 125 were excluded and 154 abstracts were read. One hundred citations were excluded and so 54 full texts were screened for inclusion. At this stage, 40 texts were excluded leaving 14 papers with results from 12 different studies to be included in the review.

It is important to acknowledge that ten studies were excluded from this review due to the limited description of NPT use. Authors provided a single statement referencing the utility of NPT in the study, with little to no further detail of its use. For example:

Individual agency and reflexive monitoring played an important part in the successful implementation of VC. [40]

Four of the ten studies were excluded as limited detail was provided to support NPT’s use in isolation of other theory/theories used in the study. For example:

Training and educational packages were developed for GPs and practice nurses by drawing on the work of May et al, Grol and Michie et al [41]

### Types of studies

In this review, 14 papers report the application of NPT in 12 studies. The data extraction table is shown in Table 3.

**Table 3:** Data extraction table

Authors	Year of study	Study type	Population/study setting/condition	Intervention	Use of NPT	Insight generated in the use of NPT	Author (of the included study) critique of NPT
Drew et al. [42]	2015	Qualitative, semi-structured interviews	**Population the intervention targets**: no specific population described other than ‘hip fracture patients’ **Setting**: Secondary care, UK (11 hospitals) **Condition**: hip fractures	Secondary fracture prevention service	**Implementation** Authors describe the aim of using extended NPT is to ‘Understand how and why secondary fracture prevention services can be implemented in secondary care’ Extended NPT was described to be used to support the development of the topic guide and analysis of the interview data (codes transposed onto the four constructs)	Four constructs of Extended NPT: (1) Capacity (2) Potential (3) Capability (4) Contribution No insight gained into the use of extended NPT. The authors only related the interview data to each of the four constructs	3 challenges in the application of extended NPT are described: 1) The overlapping nature of the constructs meaning that data could be coded into more than one construct 2) Being certain that data was categorised into the ‘correct’ construct 3) Potential for tension with undertaking an abductive analysis whilst ensuring data are not forced into pre-defined constructs
Foster et al. [43] and Saunders et al. (1) [44]	2020	Foster et al.—RCT with nested qualitative study Saunders et al.—qualitative, semi-structured interview data presented from Foster et al., RCT	**Population the intervention targets**: individuals with sciatica. RCT population: mean age 52.1 years, *n* = 476 (50% female) **Setting**: primary care, community physiotherapy and spinal interface services between primary and secondary care, UK **Condition**: Sciatica	Stratified care approach for sciatica (combines information on the risk of persistent disability with information about sciatica clinical severity)	**Implementation** NPT used alongside the ‘boundary objects concepts’ to guide qualitative study (the influence of both theoretical frameworks was clearly explained) The concept of ‘coherence’ was used as a lens to interpret the interview findings. Themes were described to be mapped onto this construct	Only ‘coherence’ construct used. No new insight gained into this construct	The authors report that the use of NPT (and boundary objects concepts) enabled the team to develop a more robust understanding of the identified issues
Gilbert et al. (1) [45]	2018	Qualitative, semi-structured interviews	**Population the intervention targets**: patients aged over 18 **Setting**: Tertiary orthopaedic hospital, UK **Condition**: Shoulder pain/dysfunction	Multiple Joint (MUJO) system (rehabilitation device)	**Implementation** NPT was used to explore the underlying reasons behind the MUJO System’s acceptability The interview schedule was described to be developed in ‘accordance with NPT’ A Direct Content Analysis was undertaken to organise the qualitative data against the NPT constructs	Four constructs used (extended NPT): (1) Capacity (2) Potential (3) Capability (4) Contribution No insight gained into construct use	Nil
Gilbert et al. (2) [46]	2019	Qualitative, semi-structured interviews	**Population the intervention targets**: patients over 18, attending the hospital for shoulder rehabilitation for shoulder instability **Setting**: Tertiary orthopaedic hospital, UK **Condition**: Atraumatic shoulder instability	Videoconferencing	**Implementation** The interview structure was based on the constructs of NPT A directed content analysis was undertaken analysing the factors in accord with NPT Coding of data was undertaken using a previously published NPT coding framework	Four constructs used (extended NPT): (1) Capacity (2) Potential (3) Capability (4) Contribution No insight gained into construct use	The authors state that ‘the use of supplementary theoretical models or a combination of open and directed content analysis might have gleaned additional data which could have informed the results’; however, no direct critique of NPT was offered
Gilbert et al. (3) [47]	2022	Qualitative, semi-structured interviews	**Population the intervention targets**: patients over age 18 years, attending the specialist orthopaedic hospital for physiotherapy or occupational therapy for an orthopaedic/musculoskeletal condition **Setting**: Tertiary orthopaedic hospital, UK **Condition**: Any orthopaedic/musculoskeletal condition	Virtual consultations (VC)	**Implementation** NPT used to underpin the line of enquiry into the implementation of VC. Preference theory also used but the use of NPT is clearly explained The interview schedule and coding frame was developed based on NPT	Four concepts used: (1) Coherence (2) Cognitive participation (3) Collective action (4) Reflexive monitoring	The authors state that ‘the use of normalisation process theory provided focused attention towards key implementation factors that feed into the formation of preference’. No other critique of the theory itself was offered
Hay et al. [48] and Ong et al. [49]	2018 2014	Hay et al.—mixed methods study NPT used in workstream 2: qualitative evaluation—observations and interviews Ong et al.—description of the conceptual underpinning of the approach in the MOSAIC study (commentary)	**Population the intervention targets**: ≥ 45 years old **Setting**: primary care, UK **Condition**: osteoarthritis	Model osteoarthritis consultation (MOSAIC)	**Implementation** The authors describe the use of NPT in 2 ways: 1) To provide a structure to identify factors facilitating implementation and overcoming barriers to delivering the intervention 2) To provide a framework for analysing and evaluating the process of intervention implementation a. Coherence construct used as an ‘organising device’ for group interviews with GPs and nurses who had undertaken intervention training b. Themes developed from data analysis were compared to the NPT construct of coherence	Although NPT is referenced to be used with all four constructs described, in the paper by Ong et al., the construct of 'coherence' is the only one mentioned	Nil
Judge et al. [50]	2016	Mixed methods study NPT used in Objective 2—Qualitative, semi-structured interviews	**Population the intervention targets**: ' hip fracture patients', no specific age given **Setting**: Secondary care (11 hospitals), UK **Condition**: hip fracture	Secondary fracture prevention service	**Implementation** The authors describe to have used extended NPT in 2 ways: 1) Interview topic guide based on four core elements 2) Codes from interview data were transposed onto the four constructs	Four constructs used (extended NPT): (1) Capacity (2) Potential (3) Capability (4) Contribution No insight gained into construct use	The authors discussed the challenge of analysing data using extended NPT with a concern of coding data into the theories constructs whilst ensuring they are not ‘forced’ into pre-defined categories. They explained to have avoided this problem by performing an initial step of inductive analysis prior to transposing the developed codes into the theory. They explained to feel that this meant any factors that did not ‘fit’ within the theory would have been identified but also felt that the theory was able to account for all of the issues relating to service implementation
Peng et al. [51]	2020	Process evaluation—qualitative, semi-structured interviews and observations	**Population the intervention targets**: ‘older hip fracture patients’, no specific age given **Setting**: Orthopaedic hospital, China **Condition**: hip fracture	Multidisciplinary co-management programme	**Implementation** The authors describe to have used NPT in 2 ways: 1) Interview guide based on NPT 2) Key themes were mapped onto the NPT constructs	Four concepts used: (1) Coherence (2) Cognitive participation (3) Collective action (4) Reflexive monitoring	The authors describe to have found the use of NPT helpful in systematically identifying barriers to implementation which allowed for consistent responses to be collected across interviews
Sanders, Foster and Ong [52]	2011	Qualitative, semi-structured interviews	**Population the intervention targets**: ‘back pain patients’ **Setting**: Primary care, UK **Condition**: back pain	Subgrouping tool	**Implementation** The authors describe the use of NPT in 3 ways: 1) Semi-structured interviews 'organised' around the 4 NPT dimensions 2) NPT used as a guiding theoretical framework for emerging themes and concepts 3) Key themes from the analysis of second stage interviews were mapped onto the 'coherence' construct	All four concepts were described to be used, with a focus on the concept of ‘coherence’. No insight generated into NPT use	The authors state to have found very little evidence that the constructs of collective action, cognitive participation and reflexive monitoring accurately reflected the behaviours and attitudes of their study population (GPs)
Saunders et al. (2) [53]	2022	Qualitative, semi-structured interviews and focus groups	**Population the intervention targets**: no clear description other than ‘employed patients consulting an FCP with MSK pain’ **Setting**: Primary care **Condition**: MSK pain	Vocational advice	**Implementation** 1) Mapping interview themes onto the four components (themes were then explored in relation to how well they ‘fit’ the four NPT components)	Four concepts used: (1) Coherence (2) Cognitive participation (3) Collective action (4) Reflexive monitoring	The authors describe the use of NPT to enable them to develop a more robust understanding of the implementation potential. Further, a strong level of ‘fit’ was described between the authors’ themes and the NPT components (i.e. none of the findings fell outside of the parameters of NPT, and the identified themes could be usefully explained through the lens of NPT)
Wylde et al. [54]	2018	Mixed methods study NPT used in stage 4—online questionnaire	**Population the intervention targets**: people with chronic knee pain after total knee replacement **Setting**: Secondary, UK **Condition**: Total knee replacement	Assessment clinic—STAR care pathway	**Implementation** 1) NoMAD instrument used to measure implementation processes from the perspectives of stakeholders	NoMAD instrument used (23-item instrument with 20 core construct items that reflect the four core constructs of NPT)	Nil
Volkmer et al. [55]	2021	Qualitative, semi-structured interviews	**Population the intervention targets**: postoperative hip fracture patients **Setting**: Secondary care, UK **Condition**: Hip fracture	Postoperative physiotherapy	**Implementation** 1) NPT drawn upon to aid interpretation of interview findings (themes)	Four concepts used: (1) Coherence (2) Cognitive participation (3) Collective action (4) Reflexive monitoring	Nil

Included articles were of a qualitative (*n* = 9) [42, 44–47, 51–53, 55] and mixed methods design (*n* = 4) [43, 48, 50, 54], with one commentary included to support the justification of NPT use in one study [49]. Among the qualitative studies, semi-structured interviews were the most common method (*n* = 8) [42, 44–47, 50, 52, 55] with two studies utilising semi-structured interviews alongside observations [51] or focus groups [53].

The studies adopting a mixed methods design incorporated multiple workstreams. In these instances (*n* = 4), only the stage incorporating NPT was extracted. Qualitative methods were used in three of the mixed methods studies (semi-structured interviews [43, 50], observations and interviews [48]) and one study used a quantitative online questionnaire [54].

### What was NPT used to support?

#### Intervention development, implementation and refinement

In all 12 studies, NPT was used to support intervention implementation. There was no evidence of its use for intervention development or refinement. However, one study reported to have used the results from the NoMAD instrument, evaluating the intervention’s implementation (in addition to results from the three other elements of the multi-phase study) to support the refinement of the intervention in further work [54].

In two studies [43, 47], NPT was used alongside another theory: boundary objects concepts [43] and preference theory [47].

#### Interventions and conditions

Of the 12 studies, five of the implemented interventions were a new clinic pathway, service or consultation [42, 48, 50, 51, 54]; two were interventions related to rehabilitation [45, 55] or an assessment tool for a specific condition [43, 52]; two were a consultation medium [46, 47]; and one was a vocational advice intervention [53].

The condition most commonly addressed by an intervention was hip fractures (*n* = 4) [42, 50, 51, 55], followed by conditions related to the shoulder (*n* = 2) [45, 46] and lower back (*n* = 2) [43, 52]. Two interventions targeted generic musculoskeletal and/or orthopaedic conditions [47, 53] and one intervention targeted a condition specific to the knee [54] and one the condition of osteoarthritis [48].

A summary is shown in Table 4.

**Table 4:**
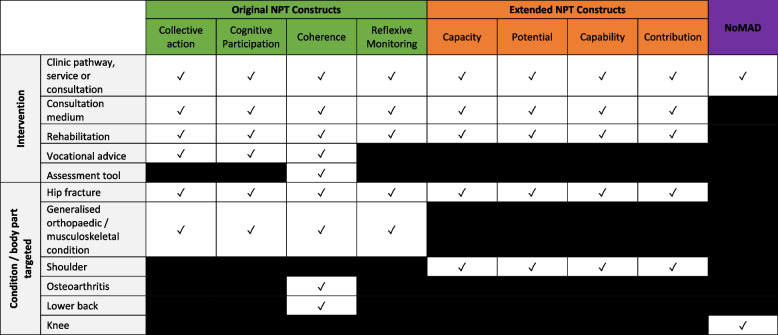
Summary of NPT use according to intervention and conditions

### NPT use, justification and insight generated

**Fig. 6:**
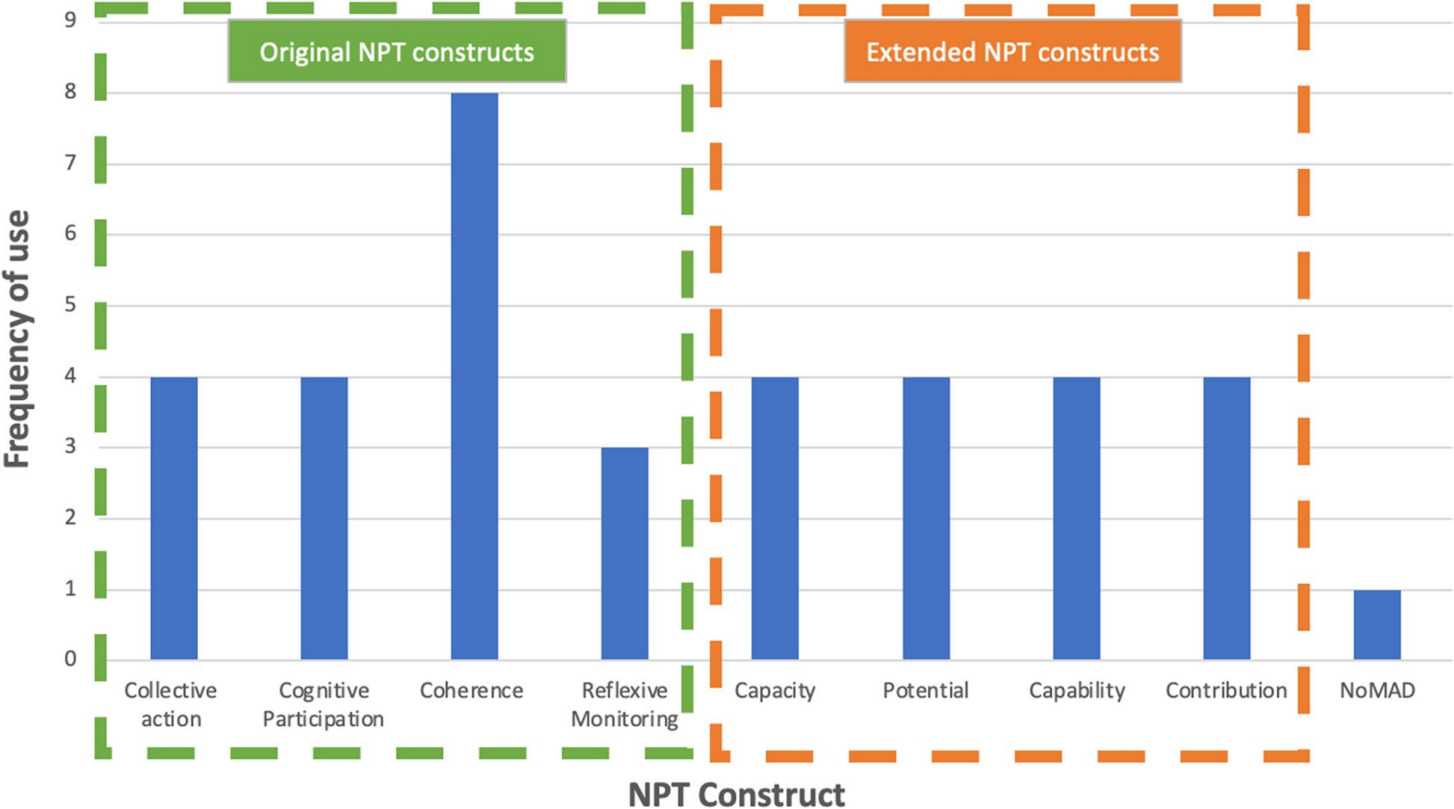
NPT construct frequency

The frequency of use for each NPT construct is summarised in Fig. 6.

The construct of coherence was the most used, cited in eight papers (7 studies) [43, 44, 47, 49, 51–53, 55]. Three studies used all four constructs of the original theory [47, 51, 55]. One study [53] reported to use all four constructs of the original theory but there was no evidence in the results of the use of reflexive monitoring. Where ENPT was used (four studies), all four constructs were used together as a ‘set’ [42, 45, 46, 50]. No study utilised any of the original constructs in combination with one or more construct from ENPT. Thirteen of the included papers were published after the introduction of the ENPT in 2013. There was limited justification across all studies for the selection of NPT and individual construct use. Instead, authors simply stated its use such as:

We used qualitative methodology to explore the underlying reasons behind the MUJO System’s acceptability and this work was informed by Normalisation Process Theory (NPT). [45]

The framework supplied by these concepts from NPT was used in two ways. First, it provided a structure for the research team in its approach to engaging with the practices by identifying factors facilitating implementation and overcoming barriers to delivering the MOSAICS intervention. Second, it provided a framework for analysing and evaluating the process of implementing the intervention. [48]

#### Coherence

This construct was suggested to be a fundamental first step in intervention implementation [43, 49, 52].

The mechanism of ‘coherence’, which is concerned with sense-making and giving meaning to a new intervention, is a pivotal first stage for implementation, and a focal point of our study. [49]

It was explained, in all studies, to support ‘sense making’ and understanding of the intervention amongst patients and/or clinicians. In three papers [43, 44, 49], it was further explained to understand the degree of meaning of the intervention. One study [52] explained to have chosen this concept alone, as they found little evidence to support the use of any other constructs for their population.

There was no new insight gained into the utility of this construct from that already reported in the literature by May and Finch [14]. All results reported under the heading of coherence, related to understanding/sense-making of the intervention by patients/clinicians.

#### Cognitive participation, collective action and reflexive monitoring

Cognitive participation and collective action were used in four studies and reflexive monitoring was used in three studies.

There was no discussion in any of the studies utilising these constructs as to the reason for their use. In all studies, results were reported against each construct, for example:

Shared decision-making and effective communication strategies were suggested as mechanisms to overcome barriers to engagement in physiotherapy, as well as the need to tailor approaches to accommodate differing individual needs (collective action). [55]

**Reflexive monitoring**

Patients were forthcoming with feedback about their experiences. [47]

The interventions being implemented in the studies that utilised these constructs were as follows: a consultation medium for patients with an orthopaedic/MSK condition [47], a multi-disciplinary care management programme for patients following a hip fracture [51], a vocational advice intervention for patients with MSK pain [53] and physiotherapy for postoperative hip fracture patients [55].

There was no new insight gained into the use of these three constructs from that already documented in the literature [14].

#### Extended NPT

The four papers citing the constructs of ENPT [42, 45, 46, 50] utilised all four concepts, with no study using one concept in isolation of the remaining three.

All four studies utilised each construct as a heading to present their results against and described these in line with the original descriptions outlined by May in 2013 [16]. Examples include:

Capacity – Social structural resources available to patients and clinicians

The accessibility of the equipment for patients and clinicians was identified as the main barrier to using the device [45]

Participants’ contributions to enacting a fracture prevention service depend on them investing in meaning, commitment, effort and appraisal.

Fracture prevention co-ordinators did not change the clinical work that was undertaken. Rather, their introduction changed the way the work was organised and delivered. Multidisciplinary meetings were used to sustain the potential and capacity of professionals involved in service delivery. [50]

The interventions being implemented in the studies that utilised these constructs were as follows: a rehabilitation device to treat shoulder pain/dysfunction [45], videoconferencing for atraumatic shoulder instability [46] and a fracture prevention service for patients following a hip fracture [42, 50].

#### NoMAD

The NoMAD instrument was used in one study [54] to assess health professional stakeholders’ views (involved in the development or future delivery of the intervention) about the implementation of a care pathway for people with chronic knee pain after knee arthroplasty. Results from the survey were collated and presented as a descriptive summary. An exemplar results statement is shown below:

Stakeholders’ opinions varied about how different the STAR care pathway was to usual patient care. This may reflect diversity in current practice for assessment, management and treatment of chronic post-surgical pain. [54]

### Critique and commentary of NPT use

Six studies (seven papers) commented on the benefits of using NPT/ENPT to:

1. Focus attention towards key implementation factors [47, 51]
2. Produce more robust understanding of identified issues [43, 44, 53]
3. Draw attention to specific elements of sense-making and community of practice [48]
4. Account for all issues relating to implementation during data analysis [50]

Negative aspects of NPT use were less frequently documented, with only two studies reporting difficulties with its use [42, 52]. One study commented on the overlapping nature of ENPT constructs and resultant uncertainty as to whether data were coded correctly.

A challenge in the application of extended Normalization Process Theory was the overlapping nature of the constructs, meaning that data could be coded into more than one construct [42]

The second study [52] described the lack of available evidence to support the use of three NPT constructs (collective action, cognitive participation and reflexive monitoring) to reflect the data for their studies population (behaviours and attitudes of GPs) and subsequently justified the use of only one construct (coherence).

We were interested in explaining the obstacles to the early adoption of the new system, which fitted in with the concept of ‘coherence’, and because we found little evidence that the other NPT constructs (for example, cognitive participation, collective action, and reflexive monitoring) accurately reflected the behaviours and attitudes of the GPs in this study. [52]

Three research teams decided to use an abductive approach to analyse data due to concern that NPT, used to develop a framework approach, may not capture all important data elements [42, 50, 53]. They described analysing data inductively as a preliminary step before mapping data onto the NPT framework to combat this concern. However, two teams [50, 53] commented that NPT was able to account for all issues relating to implementation.

Undertaking an abductive analysis, to enable us to use extended NPT, is potentially challenging, as data must be coded into constructs while it is ensured that they are not ‘forced’ into pre-defined categories. We avoided this problem by performing an initial inductive analysis to identify factors that may impact on the implementation of services and then transposing them onto the theory. Doing so meant that any factors that did not ‘fit’ within the theory would have been identified. However, we found that the theory was able to account for all of the issues relating to service implementation. [50]

One study commented that where NPT was used to support analysis, this was primarily as a guide to ensure data were not restricted.

NPT was adopted as the guiding theoretical framework underpinning emerging themes and concepts. However, NPT was used primarily to guide analysis, and not to restrict the exploration of other possible theoretical insights. [52]

Other commentary of its use was related more closely to the utility of theory in understanding implementation in complex healthcare environments rather than specific commentary about NPT.

Fracture liaison services are complex interventions, and a strength of the study is the use of NPT as a theoretical framework in order to help to understand something of the complexity of change within health services. [50]

No study utilised the original four constructs of NPT (cognitive participation, collective action, coherence and reflexive monitoring) in combination with those in the extended version (capability, capacity, potential and contribution). However, one could argue that those utilising ENPT are inadvertently including the original four constructs within the construct of contribution, although this distinction was not discussed in any other studies that utilised ENPT.

### Quality appraisal

The CASP qualitative tool was used to assess 12 of the 14 articles. Where two papers described the results of one study [43, 44, 48, 49], these were appraised concurrently.

All papers were deemed to be of sufficient quality using the CASP tool. In general, there was limited evidence in six studies [42, 50–54], to support the appraisal of question six regarding the relationship between the researcher and the participants. However, appraisal against the remainder of the questions was felt to be sufficient to not call the rigour of the articles into question. It is also acknowledged that lack of evidence to answer this question may be due to limitations with manuscript word count and reflexivity of researchers not being a formal requirement from journals for publication.

### Assessment of confidence

There were eight key findings assessed against the GRADE-CERQUAL criteria. The results of the assessment are shown in Table 5.

**Table 5:** GRADE-CERQual assessment

Summary of review finding	Studies contributing to this review finding	Methodological limitations	Coherence	Adequacy	Relevance	CERQual assessment of confidence
1. Coherence is the most commonly utilised construct to understand intervention implementation and is seen as a fundamental first step	Foster et al. [43]; Gilbert et al. [47]; Ong et al. [49]; Peng et al. [51]; Sanders et al. [52]; Saunders et al. [44]; Saunders et al. [53]; Volkmer et al. [55]	**No/Very minor concerns** (CASP scoring used to support assessment of this criterion)	**Moderate concerns** (The construct of coherence is the most frequently used construct of NPT across the dataset. However, only 3 studies [4 papers] used coherence in isolation. The remaining 4 studies therefore do not utilise coherence (construct) as a first step and so there are moderate concerns regarding coherence (assessment criterion) for this review finding)	**Moderate concerns** (The 3 studies utilising coherence as a first step offered limited rationale for this so there are concerns regarding the richness of data supporting this review finding)	**No/Very minor concerns** (8 studies of relevance to the review question, objectives and eligibility criteria. Range of interventions and MSK/orthopaedic conditions across the studies)	**Moderate confidence**
2. To date, only evidence of NPT (including ENPT) use to support the implementation of MSK/orthopaedic interventions is available and so it is unclear how NPT can be used to support intervention development and refinement	Drew et al. [42]; Foster et al. [43]; Gilbert et al. [45]; Gilbert et al. [46]; Gilbert et al. [47]; Hay et al. [48]; Judge et al. [50]; Ong et al. [49]; Peng et al. [51]; Sanders et al. [52]; Saunders et al. [44]; Saunders et al. [53]; Volkmer et al. [55]; Wylde et al. [54]	**No/Very minor concerns** (CASP scoring used to support assessment of this criterion)	**No/Very minor concerns** (The review finding is coherent with the underlying data)	**Minor concerns** (Minor concerns as although NPT is used to support implementation across all 12 studies [14 papers] there is limited discussion offering further detail and insight into its use. Data richness therefore limited)	**No/Very minor concerns** (12 studies of relevance to the review question, objectives and eligibility criteria. Range of interventions, MSK/orthopaedic conditions and healthcare settings across the studies)	**High confidence**
3. There is no evidence that the intervention or MSK/orthopaedic condition being targeted affects the utility of NPT/ENPT	Drew et al. [42]; Foster et al. [43]; Gilbert et al. [45]; Gilbert et al. [46]; Gilbert et al. [47]; Hay et al. [48]; Judge et al. [50]; Ong et al. [49]; Peng et al. [51]; Sanders et al. [52]; Saunders et al. [44]; Saunders et al. [53]; Volkmer et al. [55]; Wylde et al. [54]	**No/Very minor concerns** (CASP scoring used to support assessment of this criterion)	**No/Very minor concerns** (The review finding is coherent with the underlying data. No issues were reported in any paper regarding the suitability of NPT for MSK/orthopaedic conditions)	**Minor concerns** (No discussion in any study regarding the suitability of NPT for any MSK/orthopaedic condition. Data richness to support NPT’s use for any MSK/orthopaedic condition is limited, however data to refute its use is non-evident)	**No/Very minor concerns** (12 studies of relevance to the review question, objectives and eligibility criteria. Range of interventions, MSK/orthopaedic conditions and healthcare settings across the studies)	**High confidence**
4. It is unclear when utilisation of the original constructs of NPT may be more appropriate to those than ENPT and vice versa	Drew et al. [42]; Foster et al. [43]; Gilbert et al. [45]; Gilbert et al. [46]; Gilbert et al. [47]; Hay et al. [48]; Judge et al. [50]; Ong et al. [49]; Peng et al. [51]; Sanders et al. [52]; Saunders et al. [44]; Saunders et al. [53]; Volkmer et al. [55]; Wylde et al. [54]	**No/Very minor concerns** (CASP scoring used to support assessment of this criterion)	**No/Very minor concerns** (This review finding is coherent with the underlying data as there is limited to no discussion to support the use of NPT over ENPT and vice versa)	**No/Very minor concerns** (No discussion in any study regarding the appropriateness of either version of the theory and so there are no/very minor concerns regarding this review findings)	**No/Very minor concerns** (12 studies of relevance to the review question, objectives and eligibility criteria. Range of interventions, MSK/orthopaedic conditions and healthcare settings across the studies)	**High confidence**
5. There is limited evidence to support the NoMAD instruments’ utility	Wylde et al. [54]	**No/Very minor concerns**	**No/Very minor concerns** (only one study utilising NoMAD. The instrument is not referred to in any other study and so this review finding is coherence with the underlying data)	**Minor concerns** (Data richness to support NoMADs use is limited; however, data to refute its use is non-evident)	**Minor concerns** (1 study and therefore only 1 condition [total knee replacement], intervention type [clinic pathway] and setting [secondary care])	**High confidence**
6. NPT/ENPT is a useful analytical lens to focus researcher’s attention to understanding implementation factors more robustly and accounting for a range of identified issues	Foster et al. [43]; Gilbert et al. [47]; Hay et al. [48]; Judge et al. [50]; Peng et al. [51]; Saunders et al. [44]; Saunders et al. [53]	**No/Very minor concerns**	**No/Very minor concerns** (The review finding is coherent with the underlying data)	**Minor concerns** (7 papers discussing the usefulness of NPT in implementation however further detail to offer greater insight into its use would have been beneficial. This would have added a greater degree of richness to support this review finding)	**No/Very minor concerns** (6 studies [7 papers] of relevance to the review question, objectives and eligibility criteria. Range of interventions, MSK/orthopaedic conditions and healthcare settings across the studies)	**High confidence**
7. The application of ENPT may pose a challenge for researcher’s due to the overlapping nature of constructs	Drew et al. [42]	**No/Very minor concerns**	**No/Very minor concerns** (Although only one study supports this review finding, there were no data to challenge it. The finding is therefore coherent with the underlying data)	**Moderate concerns** (Only 1 study supporting this review finding. Further detail would have added greater insight to support the understanding of the challenged in ENPT’s application)	**Minor concerns** (1 study and therefore only 1 condition [hip fracture], intervention type [service] and setting [secondary care])	**Moderate confidence**
8. There is limited evidence that the population targeted by the intervention limits the use of particular NPT constructs	Sanders et al. [52]	**No/Very minor concerns**	**Moderate concerns** (Moderate concerns with this finding as only one study reported limited evidence to support the use of 3 NPT constructs for their population. However, no issues were reported in all other studies. An alternative explanation may be that although there was no empirical evidence supporting the constructs used in the studies’ population, it does not necessarily mean that NPT is not suitable for use)	**Moderate concerns** (Only 1 study questioned the utility of NPT (3 constructs) in a specific population. There was limited discussion as to why the authors felt the constructs didn’t reflect the population in question and evidence of this would have provided a greater depth of understanding)	**Minor concerns** (1 study and therefore only 1 condition [back pain], intervention type [assessment tool] and setting [primary care])	**Moderate confidence**

## Discussion

### Key results

In this review, we identified 12 studies utilising NPT during intervention implementation for orthopaedic/MSK conditions. These studies were reported or discussed in 14 peer-reviewed journal articles. Eight key findings were assessed using GRADE-CERQUAL with five of high and three of moderate confidence.

The use of NPT was most prevalent in qualitative study designs. It was used to support intervention implementation across primary, secondary and tertiary care settings for a range of MSK and orthopaedic conditions including specific conditions such as hip fractures, sciatica and atraumatic shoulder instability and more generically described conditions such as MSK pain. The constructs and theory (NPT/ENPT) were used in line with that which has previously been described by May and Finch [14, 16, 17].

A summary of recommendations is shown in Table 6.

**Table 6:** Summary of recommendations for NPT and ENPT based on review findings

Recommendation based on review findings	
1	NPT/ENPT can be used to support and understand implementation of orthopaedics/MSK interventions
2	Consider NPT/ENPT to support intervention development and refinement. Offer detail of its use and usability to inform future research in this area
3	NPT/ENPT seems appropriate for use across all orthopaedic/MSK conditions and target populations
4	Consider the additional constructs offered by ENPT to support greater exploration of contextual factors but be cautious of the potential challenges with the overlapping concepts. Inviting a researcher familiar with NPT/ENPT may support this
5	Report on the challenges of using NPT/ENPT to inform future researchers
6	Consider using the NoMAD tool and reporting on the instruments’ utility to add to the evidence base and inform future researchers

### Results in context

Implementation of complex interventions should be envisioned as an iterative process [3, 4, 17]. However, the predominant use of a single construct (coherence) and the suggestion that this is a fundamental first step in the implementation process is indicative that, for interventions specific to orthopaedic/MSK conditions included in this review, implementation using NPT is still seen as linear. Viewing implementation as a one-directional process is problematic. Coherence is concerned with agents perceiving a need for the intervention and seeing it as meaningful. If this is considered to be met, with no other elements explored, there is potential for research teams to miss factors that could instantly disrupt intervention adoption at the next stage, examples include:

1. Lack of resources available to support workability of the intervention (collective action)
2. Lack of outcomes recorded to evaluate the interventions use and thus lack of evidence to demonstrate its benefit to service commissioners (reflexive monitoring)

Further, if the next step is reached along this linear process, it is implied that factors relating to coherence are not revisited. As the intervention and research evolves, key issues relating to coherence may be missed that subsequently affect intervention normalisation further down the line. Therefore, considering coherence in isolation of other factors seems to narrow the practice for complex intervention implementation in health care and we encourage moving away from this linear model. We acknowledge that viewing implementation as linear may only be one account for the use of coherence as a standalone construct. However, in the absence of reflexive accounts from authors offering further insight into this, alternative explanations are limited.

In addition, the use of NPT in isolation, without the additional elements outlined in the updated ENPT, may result in key contextual factors facilitating or inhibiting the intervention being missed [3]. The context in which the intervention is to be implemented is an important consideration, given that it will exert its influence by altering existing practices, changing resource utility and impacting upon user and deliver relationships. Context is described by Skivington et al., to be considered as both dynamic and multi-dimensional to include physical, spatial, organisational, social, cultural, political or economic features [3]. However context has been acknowledged as an important but poorly understood aspect of implementation [6, 56]. This is perhaps because a universally accepted explanation of context has yet to be established, as recognised in a recent systematic review which summarised 64 studies with the aim of defining and assessing context in healthcare implementation studies [57]. The review encouraged the development of an operational definition to support consistency in future research studies and to allow for context to be appropriately accounted for. In the absence of a unified definition, the constructs offered in ENPT support the consideration of contextual elements (including social norms and roles and cognitive and material resources—capacity, individual intentions and shared commitments—potential) [16]. Whilst a single tool/theory may not capture and account for all relevant elements, lack of any consideration of the context is not supported by the current evidence base. Use of the original constructs of NPT alone could therefore be considered insufficient. However, as the studies included in this review did not report or discuss this, NPT’s utility to address contextual factors is unclear.

The use of NPT in supporting implementation was demonstrated across a range of healthcare settings for interventions targeting several different orthopaedic/MSK interventions. There was consistent stability of each NPT construct across the studies, used against their description in the literature, demonstrating NPT’s ability to be applied successfully to a range of intervention research projects. The original developers suggest that the optimal way to employ NPT is to adapt it, specific to the research goal to support workability [58]. This review supports that NPT can be used to meet the needs of a range of interventions for several different conditions in the field of MSK/orthopaedics; however, there is limited evidence to support its adaptation. There was also no evidence for NPT’s utility in intervention development despite the use of theory being highlighted as a key action in this phase [4]. Reserving NPT for use solely in the implementation phase may delay the identification of key inhibiting and/or facilitatory factors. This practice also promotes a linear stepwise process which we have previously identified to be problematic. NPT’s utility in supporting the iterative development process therefore remains hypothetical as there is currently no evidence to evaluate its use.

There was a concern in three studies that NPT, when used to support framework analysis, may not account for all captured data [42, 50, 53]. This was addressed by the introduction of a preliminary stage of inductive analysis before data was subsequently mapped on the NPT framework. However, two study teams [50, 53] reflected that NPT was able to account for all issues relating to implementation, providing reassurance for future studies that an initial inductive stage may not be necessary. However, despite the two study teams being satisfied that analysis using NPT was sufficient, it could be argued that these interpretations could be expanded upon using ENPT. As we have suggested that the original constructs may not cover all important aspects of implementation, some elements may have been missed through the sole use of NPT.

It was acknowledged by the review team that there was a lack of justification, by study authors, for NPT’s use across the included studies. Despite this, the benefits of its use were accounted for in six of the included 12 studies. Further discussion detailing reflexive decision-making would have added deeper insight and clarity for the selection of NPT amongst other theories and for individual construct use. This would support future researchers to understand the usability of NPT and the appropriateness of its choice. Negative aspects of theory use were sparsely reported. This could be due to the user-friendliness of the theory and thus minimal issues experienced or due to the limitations to manuscript publication meaning this level of detail was edited out during manuscript preparation/publication process. In addition to journal word count limitations, there is no requirement for this detail to be evidenced according to reporting guidelines such as COnsolidated criteria for REporting Qualitative (COREQ) [59] and CONSORT [60]. This may result in pitfalls of theory use encountered by one research team being avoidably repeated by another. To improve the use of theory among researchers and in the interest of research waste and efficiency, evidence of insight into its use would be beneficial. Ten studies were also excluded from this review due to the limited description of NPT use. This contributes to the argument for sufficient detail to be documented to support other researchers in utilising NPT. Finally, some healthcare research continues to be funded, conducted and published without clear consideration of underpinning theory. Without the author insight, the benefits of theory use therefore remain unclear.

### Wider evidence

This review contributes to the evidence of NPT’s use in the implementation process and can be supplemented by the work of May et al. [61] and Huddlestone et al. [20]. In May et al.’s systematic review, 130 reports of 108 NPT studies demonstrated its use to support intervention design, implementation planning and understanding of implementation, embedding and integration in feasibility studies and process evaluations [61]. All included studies utilised NPM, NPT or ENPT, with NPT most commonly reported. Similar to the present review, May et al. found that some researchers utilised the theory in a linear manner, with sense-making seen as the preliminary step. It was also concluded that critique of NPT was rare, not all included studies justified its use and typically, NPT was used as a conceptual framework for structuring study design and data analysis.

Huddlestone et al.’s systematic review explored the application of NPT specific to UK primary care settings [20]. This review included 31 papers detailing the use of the original four constructs of NPT. The authors concluded that the theory provides a flexible framework for intervention development and evaluation and supported its use in the primary care setting. Similar to this and May et al.’s review, Huddlestone et al. encouraged future NPT users to document justifications for its use as there was limited evidence of this in the included studies. There were, however, 12 categories of author reflections documented regarding NPT use, of which three were similar to that discussed in this review: (1) useful way of understanding the experience of the implementation of innovation, from multiple perspectives, (2) risk of artificially imposing (“shoehorning”) constructs onto data collection and analysis and (3) potential for cross-over of NPT constructs. The ability of NPT to compliment other theory use was also reflected upon in three studies in Huddlestone et al.’s review. Although this was not directly stated by the two research teams utilising other theory in addition to NPT in our review, it could be implied that given the research was completed and published, NPT was compatible with other theories (boundary objects concepts [43] and preference theory [47]).

### Strengths and limitations

This review contributes to the literature in supporting the use of NPT in MSK/orthopaedic intervention research. A thorough search produced 14 studies for discussion which covered a range of interventions and conditions. Non-English articles were included in the search which widened our scope; however, none were found to be appropriate for inclusion. Although a comprehensive search was undertaken in two bibliographic databases and one search engine, it is possible that some studies were missed. The use of Google Scholar was also challenging as it produced multiple versions of the same reference creating additional work in eliminating duplications. We chose to narrow the context of intervention in the field of MSK/orthopaedic conditions which potentially reduces the transferability of results to other areas of healthcare. Although framework analysis was used and analysis using this method is likely repeatable by another research team, it is possible different conclusions could be drawn from the data by other researchers due to their experience and understanding of NPT and background. The robustness of our findings has been maximised by refining the a priori matrices with a formal review after 25% of the data were chartered and with continual discussions of the results amongst the team. The research team includes those with experience of using NPT in addition to those with minimal experience; this ensured the process was transparent and comprehensible.

### Next steps

In the field of MSK/orthopaedic conditions, researchers need to consider utilising NPT/ENPT in an iterative intervention implementation process, with multiple feedback loops rather than a linear stepwise approach. Further, attention to context is important to ensure key factors are not missed. Deeper insight into the usability of NPT is encouraged to support transparency of its use.

As defined in objective (1) of the review, we aimed to identify the use of NPT in the process of intervention development, implementation and/or refinement. In this review, NPT’s utility has only been identified in the implementation phase. The use of NPT to support intervention development and refinement therefore remains unknown and further research is needed to determine its benefit in orthopaedic/MSK research.

## Conclusion

The use of NPT/ENPT to support intervention development and refinement among the orthopaedic/MSK evidence base is sparse. Reviewing the theory’s utility in implementation has demonstrated its potential in supporting these processes and we advocate its use in future research.

The construct of coherence appears most popular with limited insight into construct selection. The specific benefits of using NPT/ENPT over another or no theory are limited, and further work is needed to define this. As context is a key factor in the intervention development and implementation process, the use of NPT alone is perhaps no longer sufficient. NPT’s utility in understanding contextual factors was unclear in the orthopaedic/MSK studies included in this review. However, evidence of the additional benefits of ENPT use is sparse. NPT/ENPT appears suitable for implementation research across a range of healthcare settings and for differing types of interventions targeting several different orthopaedic/MSK conditions. We encourage future researchers to offer clear justification for NPT’s use in their methodology.

### Supplementary Information

**Additional file 1.**

**Additional file 2.**

## Data Availability

The datasets used and/or analysed during the current study are available from the corresponding author on reasonable request.

## Abbreviations

COREQ: COnsolidated criteria for REporting Qualitative
ENPT: Extended normalisation process theory
GRADE-CERQual: Confidence in Evidence from Reviews of Qualitative research
iSoQ: Interactive Summary of Qualitative Findings
MSK: Musculoskeletal
MRC: Medical Research Council
NoMAD: Normalisation Measure Development
NPM: Normalisation process model
NPT: Normalisation process theory
