# Supplementary material for: Using normalisation process theory for intervention development, implementation and refinement in musculoskeletal and orthopaedic interventions: a qualitative systematic review
Source: Implement Sci Commun. 2023 Sep 18;4:114. doi: 10.1186/s43058-023-00499-z (PMC10506319; doi:10.1186/s43058-023-00499-z)
Supplement: Supplementary file 2 — Additional file 2. [file 43058_2023_499_MOESM2_ESM.docx]

## Additional File 2

**Citations searched (listed in order of publication)**

1. May C. A rational model for assessing and evaluating complex interventions in health care. BMC Health Serv Res. 2006;6(86):1–11
2. May C, Finch T, Mair F, Ballini L, Dowrick C, Eccles M, Gask L, MacFarlane A,Murray E, Rapley T, et al. Understanding the implementation of complex interventions in health care: the normalization process model. BMC HealthServ Res. 2007;7:148.
3. May CR, Mair FS, Dowrick CF, Finch TL. Process evaluation for complex interventions in primary care: understanding trials using the normalization process model. BMC Fam Pract. 2007;8
4. May C, Finch T. Implementation, embedding, and integration: an outline of normalization process theory. Sociology. 2009;43(3):535–54.
5. May C, Mair FS, Finch T, MacFarlane A, Dowrick C, Treweek S, Rapley T,Ballini L, Ong BN, Rogers A, et al. Development of a theory of implementation and integration: normalization process theory. ImplementSci. 2009:4(29)
6. Murray E, Treweek S, Pope C, MacFarlane A, Ballini L, Dowrick C, Finch T,Kennedy A, Mair F, O'Donnell C, et al. Normalisation process theory: a framework for developing, evaluating and implementing complex interventions. BMC Med. 2010;8(1):63.
7. May C, Finch T, Ballini L, MacFarlane A, Mair F, Murray E, Treweek S, RapleyT. Evaluating complex interventions and health technologies using normalization process theory: development of a simplified approach and web-enabled toolkit. BMC Health Serv Res. 2011;11(1):245

[www.normalizationprocess.org](http://www.normalizationprocess.org)

1. Finch T, Mair F, O'Donnell C, Murray E, May C. From theory to ‘measurement’ in complex interventions: methodological lessons from the development of an e-health normalisation instrument. BMC Med Res Methodol. 2012;12(1):69.
2. May C. Towards a general theory of implementation. Implement Sci. 2013;8(1):18
3. May C. Agency and implementation: understanding the embedding of healthcare innovations in practice. Soc Sci Med. 2013;78(0):26–33
4. Finch TL, Rapley T, Girling M, Mair FS, Murray E, Treweek S, McColl E, SteenIN, May CR. Improving the normalization of complex interventions: measure development based on normalization process theory (NoMAD): study protocol. Implement Sci. 2013;8
5. May CR, Albers B, Bracher M, Finch TL, Gilbert A, Girling M, Greenwood K, MacFarlane A, Mair FS, May CM, Murray E, Potthoff S, Rapley T. Translational framework for implementation evaluation and research: a normalisation process theory coding manual for qualitative research and instrument development. Implement Sci. 2022 Feb 22;17(1):19. doi: 10.1186/s13012-022-01191-x. PMID: 35193611; PMCID: PMC8861599.
